# IL-17 expression by breast-cancer-associated macrophages: IL-17 promotes invasiveness of breast cancer cell lines

**DOI:** 10.1186/bcr2195

**Published:** 2008-11-17

**Authors:** XingWu Zhu, Lori A Mulcahy, Rabab AA Mohammed, Andrew HS Lee, Hester A Franks, Laura Kilpatrick, Acelya Yilmazer, E Claire Paish, Ian O Ellis, Poulam M Patel, Andrew M Jackson

**Affiliations:** 1 Academic Unit of Clinical Oncology, University of Nottingham – City Hospital Campus, Nottingham NG5 1PB, UK; 2 Department of Histopathology, University of Nottingham – City Hospital Campus, Nottingham, NG5 1PB, UK; 3 Pathology Department, Faculty of Medicine, Assiut University, Assiut, Egypt

## Abstract

**Introduction:**

IL-17 plays an important role in autoimmunity, promoting autoimmunity, inflammation and invasion in multiple sclerosis, rheumatoid arthritis and type I diabetes. The role of IL-17 in cancer is unclear, however, as there are few studies examining IL-17 protein expression in cancer. We therefore examined IL-17 protein expression in human breast cancer and modelled its potential biological significance *in vitro*.

**Methods:**

Immunohistochemistry was used to determine IL-17 expression in breast cancers. Matrigel invasion assays were employed to examine the effect of IL-17 on cancer cell invasion by a panel of breast cancer cell lines. The role of matrix metalloproteinases (MMPs) was investigated with selective antagonists and immunoassays for MMP-2, MMP-3, MMP-9 and tissue inhibitor of MMP.

**Results:**

IL-17-expressing cells with macrophage morphology were identified in the peritumoural area of a proportion of patients (8/19 patients). Macrophages were confirmed by CD68 staining on serial sections. With the exception of occasional lymphocytes, one patient with rare multinucleate giant cells and one patient with occasional expression of IL-17 in tumour cells, no other IL-17-positive cells were detected. Addition of IL-17 to cell lines *in vitro *stimulated marked invasion of Matrigel. In contrast, IL-17 did not promote the invasion of MCF7 or T47D cell lines. Invasion was initially thought to be dependent on MMPs, as evidenced by the broad-spectrum MMP inhibitor GM6001 and selective antagonists of MMP-2/MMP-9 and MMP-3. Measurement of MMP-2, MMP-3 and MMP-9, and tissue inhibitor of MMP 1 secretion, failed to reveal any changes in expression following IL-17 exposure. In contrast, TNF promoted secretion of MMPs but IL-17 did not augment TNF, indicating that IL-17 acts via an independent mechanism.

**Conclusions:**

The present study is the first to describe *in situ *expression of IL-17 protein in human breast tumours and to propose a direct association between IL-17 and breast cancer invasion. The precise effectors of IL-17-dependent invasion remain to be characterised but could include a range of proteases such as a disintegrin and metalloproteinase protein or astacins. Nevertheless, this work identifies a novel potential mechanism for breast cancer invasion and tumour progression, the prognostic implication of which is currently under investigation.

## Introduction

In recent years IL-17 has become regarded as a key mediator at the interface between adaptive and innate immunity. IL-17 plays a critical role in host defence and is important in inflammatory and autoimmune diseases, including inflammatory bowel disease [1], multiple sclerosis [2] and rheumatoid arthritis [3]. Perhaps surprisingly, despite the role of IL-17 in autoimmunity, relatively little is known about its role in malignancy – and the data obtained so far are somewhat conflicting. Some reports show that IL-17 supports tumour growth, probably by stimulating angiogenesis of human cervical cancer and murine fibrosarcoma cells when transfected with IL-17 cDNA [4-6]. In contrast, other studies suggest that IL-17 promotes T-cell-mediated tumour rejection [7-9]. One recent study showed that IL-17 increases the invasive capacity of the JEG-3 human choriocarcinoma cell line [10], but the mechanisms of action remain unclear. Importantly, as previous studies have largely focused on IL-17 mRNA, there is a paucity of studies examining the expression of IL-17 protein in human malignancy.

Histological inflammation is associated with poor prognosis and a higher incidence of metastasis in breast cancer [11,12]. Given the patterns of local invasion and the inherent potential for metastasis associated with the natural history of this disease, it is believed that local inflammatory cytokines, matrix metalloproteinases (MMPs) and vascular endothelial cell growth factor play key roles [13]. Previous studies have reported that tumour-associated macrophages are a major component of the lymphoreticular infiltrates of tumours [14], and high numbers of tumour-associated macrophages are observed in many tumours including invasive breast cancer [15]. Furthermore the extent of macrophage infiltration correlates positively with angiogenesis and negatively associates with prognosis in breast cancer and malignant melanoma [16,17]. Such macrophages are thought to express higher levels of inflammatory cytokines (for example, TNF, epidermal growth factor and vascular endothelial growth factor), promoting angiogenesis, tumour growth and invasion [18-21]. The present study examined the expression of IL-17 in breast cancer and identified it as a novel candidate for inflammation-associated cancer invasion in breast cancer.

Breast cancer is among the four most common human cancers [22]. Cancer cells need to invade the surrounding extracellular matrix to gain entry into the lymphatic and vascular systems for dissemination to distant sites in the body. In this regard, proteases such as MMPs are key effectors in these processes. Elevated levels of tumour-derived MMP-9 and mononuclear inflammatory cell-derived MMP-1/MMP-7/MMP-9/MMP-11/MMP-13/MMP-14 are significantly associated with higher rates of distant metastases in breast cancer [23]. Production and activation of MMPs is dependent on various cytokines, including TNFα and IL-1 secreted by tumour cells [18,19,21], fibroblasts [24-26] and macrophages [27]. IL-17 stimulates TNFα and IL-1 production by monocytes and macrophages [28], and upregulates MMP-9 production from macrophages [29]. Previous studies found that MMPs, IL-1 and TNF are regulated by IL-17 in periodontitis [30], and found that IL-17 receptor deficiency results in impaired expression of IL-1 and MMP-3/MMP-9/MMP-13 in rheumatoid arthritis [31], indicating that IL-17 also plays an important role in the regulation of MMPs.

To date there is a paucity of published evidence on IL-17 protein expression in human malignancy, including descriptions of its cellular source and its direct effect on breast cancer cells. In the present study, we identify macrophages as a major cellular source of IL-17 in breast tumours and we show that IL-17 directly promotes breast cancer cell invasion *in vitro*. The clinical significance of this novel pathway of cancer cell invasion is currently under investigation; however, IL-17 may represent an attractive target of potential prognostic or therapeutic value.

## Materials and methods

### Reagents

Recombinant human IL-17 and recombinant TNFα were obtained from R&D Systems (Abingdon, Oxfordshire, UK). A goat polyclonal anti-human IL-17 antibody and corresponding neutralising peptide were obtained from Santa Cruz Biotechnology (Insight Biotechology, London, UK). A broad-spectrum MMP inhibitor (GM6001) and selective antagonists of MMPs (MMP-3 inhibitor II, MMP-2/MMP-9 inhibitor I) were obtained from Calbiochem (Invitrogen, Paisley, UK). Results of the immunohistochemistry and Matrigel analyses were determined using a Nikon microscope (Eclipse E600, Fujitsu Ten, Kobe, Japan).

### Specimens and immunohistochemistry

**Figure 1:**
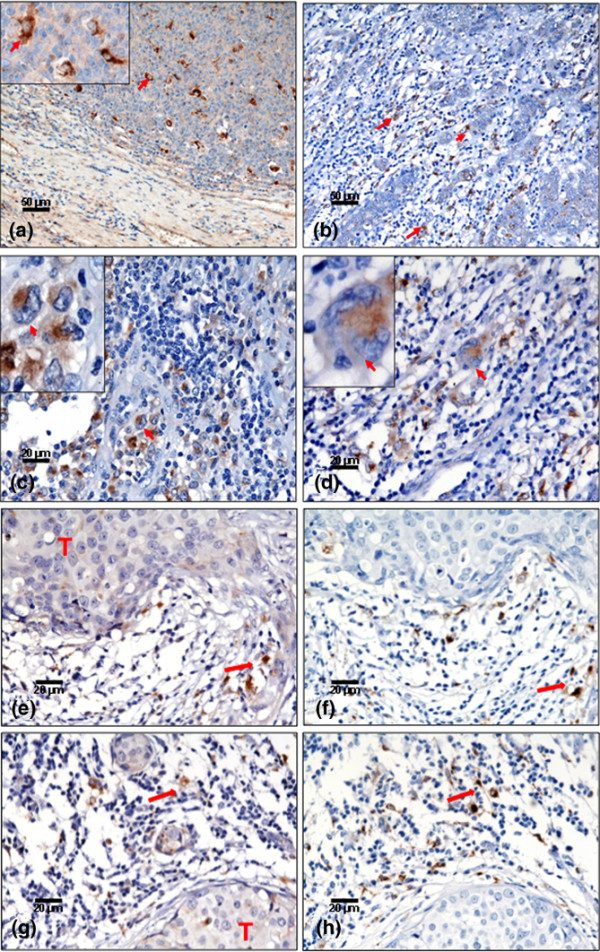
**Immunohistochemical staining of IL-17-positive macrophages in breast cancer**. **(a) **Tonsil section stained with IL-17 showing IL-17-positive cells: the shape and distribution of these cells indicate they are the macrophages within the germinal centre of the lymphoid follicles (magnification ×200). Staining of IL-17-positive cells in sections from breast cancer: **(b) **IL-17-positive cells located in the peritumoural areas (magnification ×200); **(c) **IL-17-positive macrophages characterised by having an oval or irregular shape, a kidney-shaped nucleus and a prominent nucleolus located at the nuclear membrane (arrows and inset) (magnification ×400) – this is a typical example of the level of inflammation scored as 2+; and **(d) **peritumoural area with dense inflammatory infiltrate showing one giant cell – a large-sized cell with multiple nuclei (arrow & inset) (magnification ×400). In all positive cells, staining was found to be only cytoplasmic and the nuclei are completely negative. **(e), (f), (g), (h) **Examples of IL-17 and CD68 staining in consecutive sections of breast cancer. Because it was not possible to satisfactorily stain IL-17 and CD68 on the same section, we stained consecutive sections for each antigen. As can be seen, whilst the tumour (T) is negative, the IL-17-positive cells in the surrounding stroma were found in similar areas to the CD68^+ ^macrophage (red arrow).

To determine whether IL-17-producing cells were present in breast cancer, archival paraffin-embedded sections of 19 primary invasive breast tumours (15 Grade III and four Grade II) were obtained from the Department of Histopathology, City Hospital, Nottingham, UK. The density of the inflammatory infiltrate was examined in those sections, and was scored using a semiquantitative system that recorded a value of 0 for lack of inflammation, 1+ for mild inflammation and 2+ for marked inflammation. Figure 1c shows an example of 2+ inflammation. Eleven specimens showed no inflammation, four specimens showed mild (1+) inflammation, and four specimens showed dense (2+) inflammation (Table 1). Two independent histopathologists (RAAM and AHSL) examined the staining pattern in a blinded matter. Ethical approval was obtained for the analysis, from the Nottingham Local Research Ethics Committee (REC C2020313).

**Table 1:** Summary of the immunohistochemical staining of IL-17 in breast cancer

Grade (number of patients)	Inflammatory infiltrate	IL-17-positive cells	Lymph-node-positive	Lymph vascular invasion
Grade III (n = 15)	4+, 3++	8	7 (4)	7 (5)
Grade II (n = 4)	1++	0	2	2
Total (n = 19)	8	8	9 (4)	9 (5)

Paraffin-embedded sections from 19 patients were stained and analysed. +, mild inflammation; ++, dense inflammation. Data in parentheses are IL-17-positive cells.

The staining of IL-17 antibody on paraffin-embedded sections was first optimised on tonsil sections (enlarged hyperplastic tonsil from tonsillectomy). Briefly, 4 μm sections were deparaffinised in xylene and then rehydrated in a graded sequence of ethanol solutions. For antigen retrieval, sections were pretreated in ethylenediamine tetraacetic acid buffer (p H9) in a microwave (800 W, 10 min high power, 10 min low power). After cooling, nonspecific binding was blocked with diluted serum (goat IgG ABC kit; Vectastain (Vector Laboratories, Peterborough, Cambridgeshire, UK) followed by incubation with goat polyclonal anti-human IL-17 antibody (diluted 1:100 in blocking serum) at room temperature in a humidified chamber. As a negative control, sections were incubated with normal goat IgG or the primary antibody was preincubated with a neutralising specific peptide that completely abrogated staining (Figure 1). After incubation with the primary antibody, sections were washed with PBS and subsequently treated using the goat IgG ABC kit according to the manufacturer's protocol. Peroxidase activity was visualised using the 3,3'-diaminobenzidine kit (Vector Laboratories), and sections were counterstained with haemotoxylin. Staining was examined by specialist consultant breast histopathologists from the Department of Histopathology, City Hospital, Nottingham, UK.

### Cell lines and culture

A panel of breast cancer cell lines was used for *in vitro *studies. The adenocarcinoma cell line MCF-7 and the ductal carcinoma cell line MDA-MB435 were cultured in RPMI-1640 (Sigma-Aldrich, Poole, Dorset, UK) plus 10% FCS (Sigma). The T47D ductal carcinoma cell line was cultured in RPMI-1640, 10% FCS and 0.1 μM 2-mercaptoethanol (Sigma), whilst the adenocarcinoma cell line MDA-MB231 was cultured in MEM (Sigma), 10% FCS, L-glutamine (Sigma) and nonessential amino acid solution (Sigma). All cells lines consistently screened negative for mycoplasma.

### Matrigel invasion assay

Six-well Matrigel Invasion Chambers (BD Biosciences, Oxford, UK) were used to study the invasiveness of tumour cell lines. Depending on the cell line used, RPMI-1640 or MEM supplemented with 10% FCS was added to the lower wells of the chamber. Cells were resuspended at 1.25 × 10^5^/ml (total 2 ml) in appropriate serum-free media supplemented with 0.1% BSA (Sigma), and were added to the upper compartment of the chamber. After settling, cells were treated with IL-17, TNFα or IL-17 plus MMP-3 inhibitor II/MMP-2/MMP-9 inhibitor I (1 μM) or GM6001 (100 μM). Unstimulated control inserts were always included. Plates were incubated for 22 hours at 37°C, following which noninvading cells were removed from the upper surface of the transwell membrane using a cotton swab. Transwell filters were then fixed in 100% methanol for 2 minutes and then stained in Toluidine Blue solution (Sigma) for 2 minutes before washing in water.

Inserts were excised and mounted onto microscope slides. Images of cells from three representative fields were captured digitally and the number of cells present on the transwell was counted. Results are presented as the average of triplicate determinations and each experimental condition was repeated on at least three separate occasions.

### Assay for IL-17, TNFα, MMPs and tissue inhibitor of matrix metalloproteinase 1

TNFα was measured using the Bio-Rad Luminex system (Hemel Hempstead Hertfordshire, UK). Culture supernatants were incubated with TNFα antibody bead concentrate, followed by biotinylated detector and streptavidin-RPE using the Biosource extracellular protein buffer kit (London, UK). Standard curve and interpolated results were calculated with Bioplex manager 3.0 software (Bio-Rad Laboratories, Hercules, CA, USA).

MMP-2, MMP-3 and MMP-9 were assayed in the cell-free supernatants of breast cancer cell lines using the Luminex assay as per manufacturer's instructions (R&D Systems). Levels of the tissue inhibitor of matrix metalloproteinase (TIMP)-1 and TIMP-2 tissue inhibitors were assayed by ELISA according to the manufacturer's instructions (R&D Systems).

Secretion of IL-17A was measured in standardised supernatants collected from the panel of breast cancer cell lines. Cells were seeded in 24-well plates (10^5^/ml) and were cultured for 48 hours. Cell-free supernatants were harvested, and the secreted IL-17A was determined using a modified DuoSet ELISA assay (R&D Systems). This ELISA does not react with IL-17B, IL-17C, IL-17D, IL-17E or IL-17F and has a sensitivity of 15 pg/ml for IL-17A.

### Statistical analysis

The statistical analysis on breast cancer cell invasion was performed using a Student *t *test provided in SPSS software (Chicago, Illinois, USA).

## Results

### Expression of IL-17 in breast tumours

To date there have been no published descriptions of IL-17 protein expression and localisation in breast cancer. We therefore undertook to stain a series of breast tumours for IL-17 using a modified immunohistochemistry technique. Initial staining of control tonsil sections showed scattered IL-17-positive cells (Figure 1a) and this staining was completely blocked with a specific neutralising peptide, thus confirming the specificity of the antibody (data not shown). Immunohistochemical staining was examined by a consultant histopathologist, and the morphology and distribution of these cells indicated they were macrophages located within the germinal centre of the lymphoid follicles and in the interfollicular areas.

In eight out of the 19 stained breast tumours (Table 1) we observed the presence of IL-17 strongly positive cells within the scattered tumour-associated inflammatory infiltrate. These IL-17-positive cells were located mainly in the peritumoural areas and associated with pronounced mononuclear cell infiltration (Figure 1b). The majority of IL-17-expressing cells (>98%) exhibited classical macrophage morphological characteristics, including an oval or kidney-shaped medium-sized nucleus, a prominent nucleolus located at the nuclear membrane, fine chromatin and abundant cytoplasm (Figure 1c). Positive staining was restricted to the cytoplasm and no nuclear staining was observed. None of the tumour cells stained for IL-17 expression; however, a small number of lymphocytes and an occasional plasma cell showed cytoplasmic staining. In one patient's tumour, occasional multinucleate giant cells also showed cytoplasmic staining (Figure 1d).

**Figure 2:**
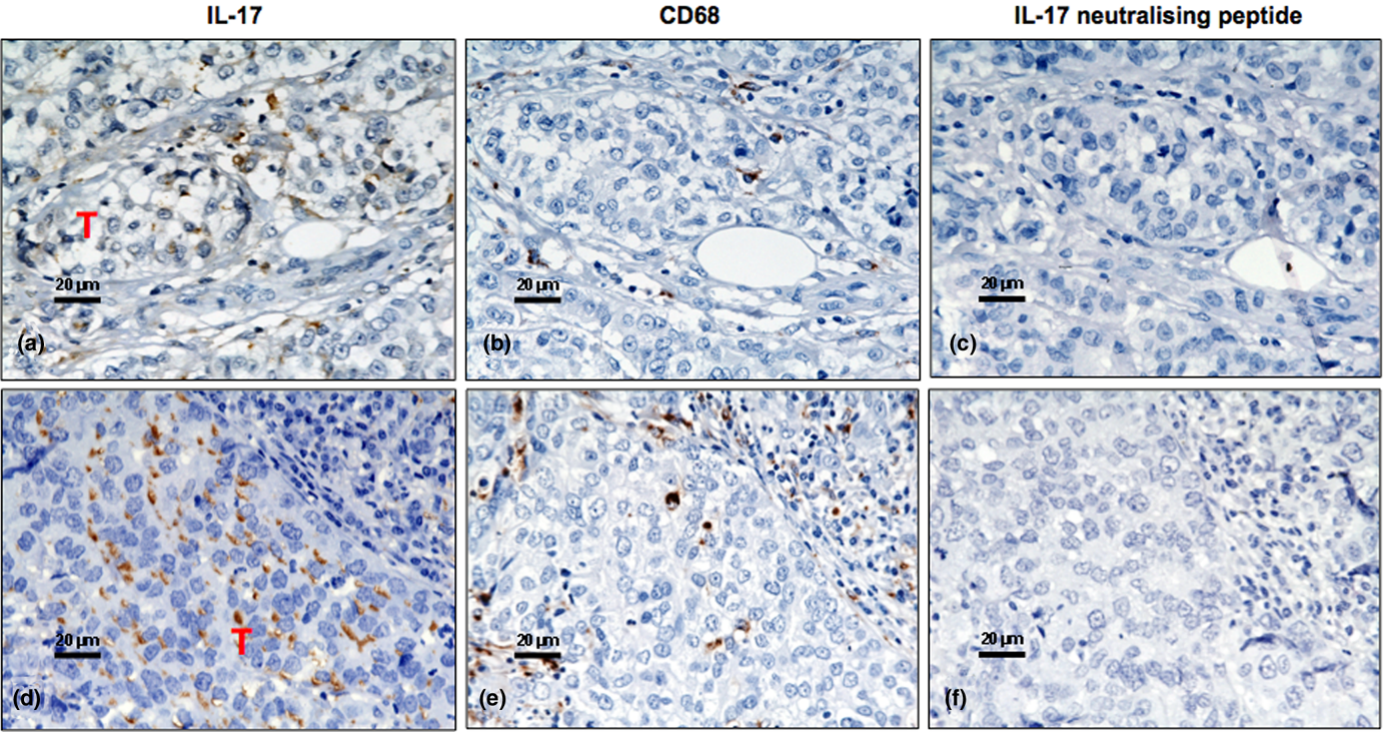
**Expression of IL-17 in breast cancer**. **(a) to (c), (d) to (f) **Consecutive sections from two patients stained for (a), (d) IL-17 and (b), (e) the CD68 macrophage marker. (c), (f) A further consecutive section showing the staining obtained when the IL-17-specific antibody was preincubated with a specific neutralising peptide. (d) Of interest, in one out of 19 patients studied there were occasional areas of tumour (T) that stained specifically with the anti-IL-17 antibody.

It was not possible to obtain satisfactory dual staining for intracellular IL-17 and the macrophage marker CD68 on archival material. To further demonstrate the origin of the IL-17-positive cells we stained consecutive sections for the macrophage marker CD68. Cells that were positive for CD68 occurred in the same area of the tumour material and, as expected, displayed similar morphological characteristics as IL-17-positive cells (Figure 1e to 1g). Staining of IL-17 was observed in a fraction of the tumour cells from only one patient (Figure 2d). Whilst there were occasional intratumoural macrophages (Figure 2e), these were not in sufficient abundance to account for IL-17 staining in tumour cells *per se*. The specificity of IL-17 staining was confirmed by the preincubation of antibody with the specific neutralising peptide, which resulted in almost complete ablation of signal (Figure 2c,f).

To further explore this observation, we analysed the secretion of IL-17 from a panel of breast cancer cell lines including the MDA-MB435 cell line – which, although frequently described as of mammary adenocarcinoma origin, may actually originate from metastatic melanoma. None of the cell lines studied constitutively secreted detectable levels of IL-17 (data not shown).

### IL-17 drives invasiveness of breast cancer cell lines *in vitro*

**Figure 3:**
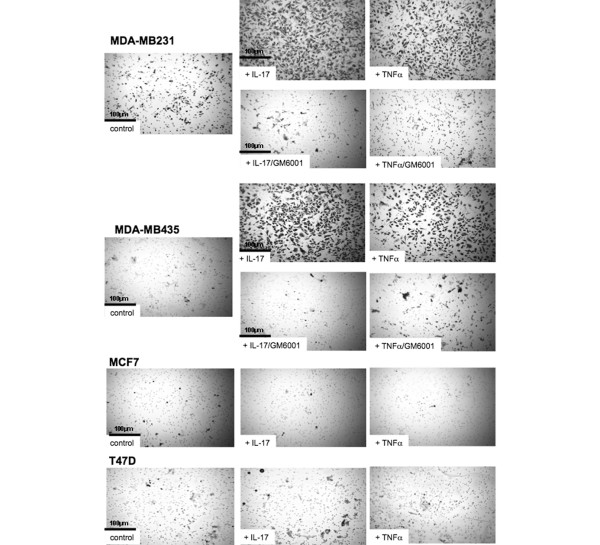
**IL-17 promotes the invasiveness of breast cancer cell lines**. Representative micrographs (magnification ×200) of Transwell filters following the invasion of the four indicated tumour cell lines either resting, treated with IL-17 (100 ng/ml), treated with TNFα (40 ng/ml) or in the additional presence of a pan-matrix metalloproteinase inhibitor GM6001 for 22 hours. Because of the absence of invasion for the MCF7 and T47D cell lines, the micrographs in the additional presence of matrix metalloproteinase inhibitor are not included.

**Figure 4:**
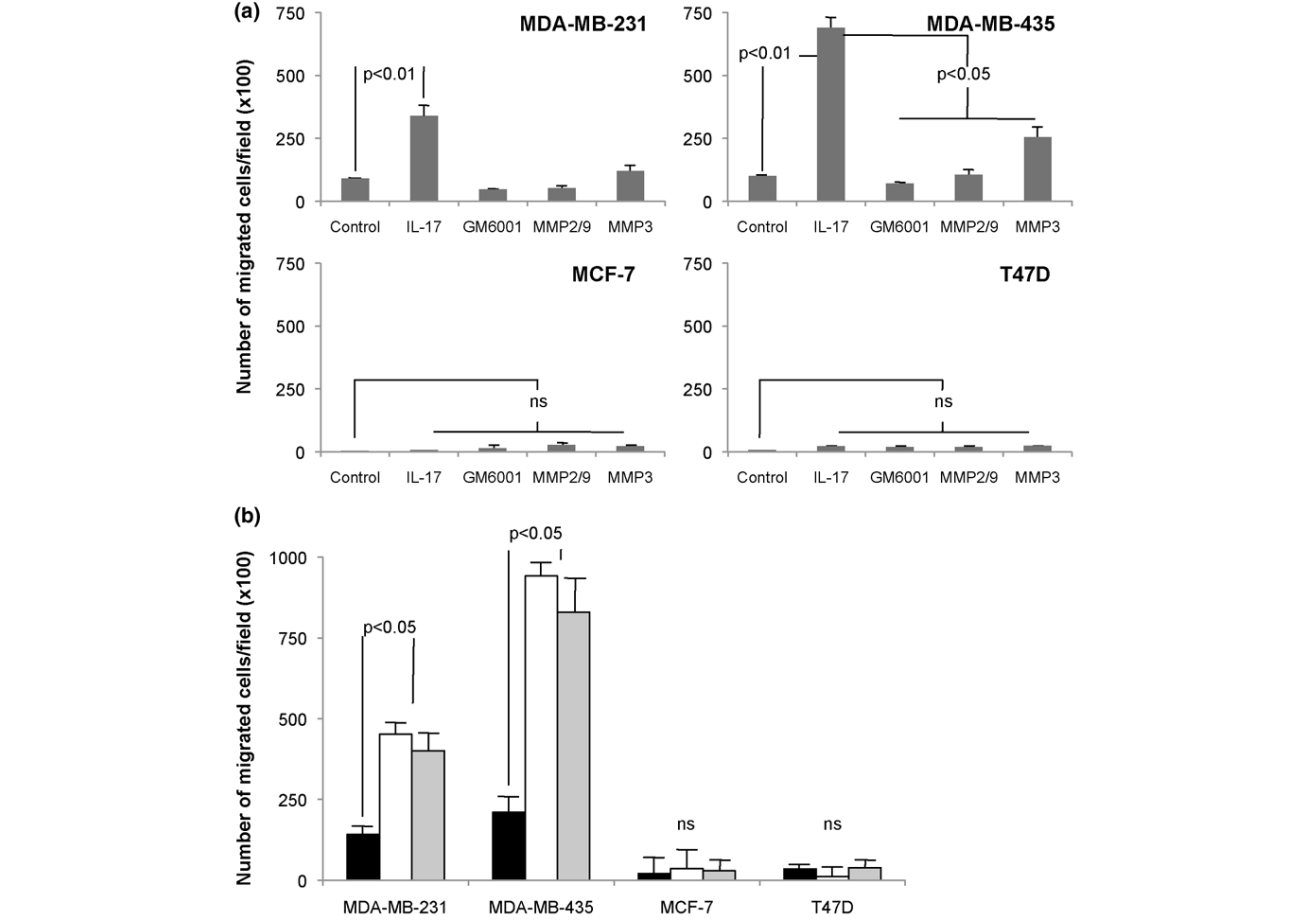
**IL-17 promotes invasion of Matrigel**. **(a) **IL-17 stimulates breast cancer cell invasion and is suppressed by selective matrix metalloproteinase (MMP) antagonists as indicated. Breast tumour cell lines were incubated in serum-free conditions in the absence or presence of IL-17 (200 ng/ml) ± MMP antagonists on the upper chamber of a Matrigel-coated transwell for 22 hours at 37°C. The Matrigel layer was removed and the cells on the transwell were stained prior to mounting, imaging and enumeration. The number of cells migrated onto the transwell per field (magnification ×100) were counted, three representative fields for each condition were enumerated, and each condition was repeated at least three times in separate experiments. Statistical significance is indicated where appropriate. ns, not significant. **(b) **Comparison of the proinvasive effect of IL-17 (200 ng/ml, white bar) versus TNFα (40 ng/ml, grey bar) versus untreated cells (black bar) on a panel of four breast cancer cell lines. Invasion assays were conducted as described above. Data represents the mean ± standard deviation of triplicate determinations and is representative of five independent experiments. Statistical significance is indicated.

Because of the observed peritumoural pattern of IL-17 staining in breast cancer and the known contribution of macrophages to tumour invasion [27,32], we examined the influence of IL-17 on the invasive capacity of a panel of breast cancer cell lines. Addition of IL-17 to the MDA-MB231 and MDA-MB435 cell lines promoted marked and significant invasion of Matrigel (Figures 3 and Figure 4). Breast cancer invasion has previously been shown to be strongly promoted by TNFα, and in our experiments IL-17-dependent invasion was of similar magnitude to that achieved following stimulation with TNFα. In contrast, the MCF-7 and T47D cell lines, classically regarded as noninvasive, had no intrinsic invasive capacity, and did not invade Matrigel in response to either TNFα or IL-17 (Figures 3 and 4).

### IL-17-dependent invasion of breast cancer cell lines is inhibited by selective-antagonists of MMP-2, MMP-3 and MMP-9

Invasion of Matrigel is thought to require the presence and activation of MMPs [27]. We therefore examined the role of MMPs in IL-17-dependent invasion of Matrigel using a panel of selective MMP antagonists. Addition of the pan-MMP inhibitor GM6001 (inhibitor of MMP-1, MMP-2, MMP-3, MMP-8 and MMP-9) caused a significant inhibition of IL-17-dependent invasion (*P *< 0.001). Furthermore, selective antagonists for MMP-2/MMP-9 or MMP-3 also suppressed the stimulatory effect of IL-17 on breast cancer invasion, although to a lesser extent than GM6001 (Figure 4). Although significantly inhibited, the MMP-3 selective antagonist was the least effective at reducing the invasiveness of the MDA-MB231 and MDA-MB435 cell lines.

**Figure 5:**
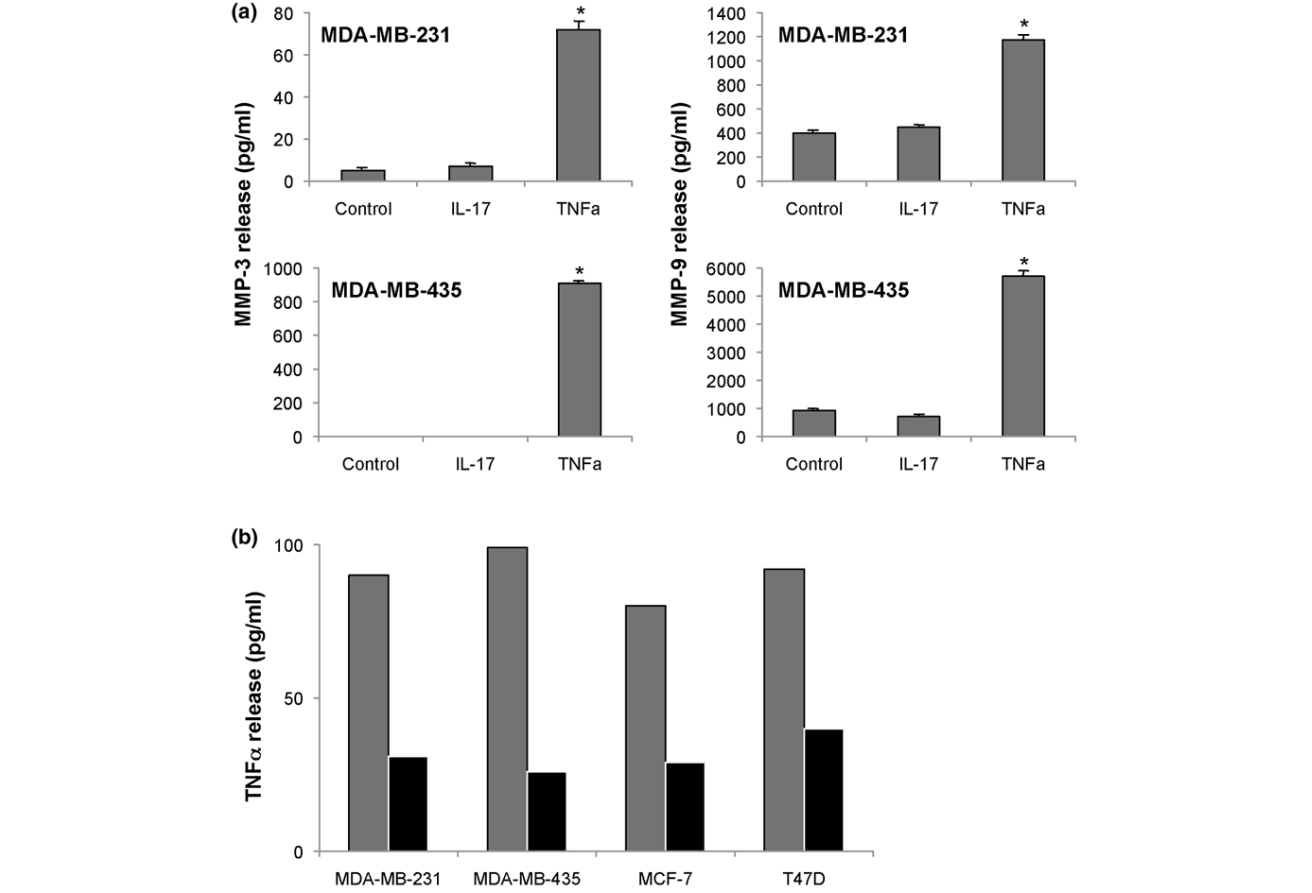
**Effect of IL-17 on MMP and TNF expression**. **(a) **IL-17 does not affect the secretion of matrix metalloproteinase (MMP)-2, MMP-3, MMP-9 or tissue inhibitor of matrix metalloproteinase (TIMP). The supernatants of control and treated (IL-17 or TNFα) tumour cell lines were assayed for the presence of MMP-2, MMP-3, MMP-9 and TIMP-1 and TIMP-2 by Luminex immunoassay or ELISA. The invasive breast cancer cell lines MDA-MB231 and MDA-MBA435 were seeded into 24-well plates (1 × 10^5^/well) and rested overnight. Cells were treated with IL-17 (200 ng/ml) or TNFα (40 ng/ml) for 24 hours and the supernatants were harvested for assay. Results are representative of two independent assays and show the mean ± standard deviation of triplicate determinations for one of two experiments (*P < 0.01). The levels of MMP-2 were unaffected by any treatment, and the levels of TIMP inhibitors remained unchanged (data not shown). **(b) **IL-17 acts independently of TNFα. A panel of breast cancer cell lines were seeded into 24-well plates (1 × 10^5^/well) and rested overnight. Cells were left untreated (grey bar) or were treated with 200 ng/ml IL-17 (black bar) for 24 hours and the supernatants were harvested for TNFα ELISA. Results are representative of two independent assays and show the mean ± standard deviation of triplicate determinations for one of two experiments (*P < 0.01). The concentration of recombinant added TNFα (expected 40 ng/ml) required to stimulate migration was actually found to correspond to 17,205 ± 1,020 pg/ml in this assay.

To further address a possible role for MMPs in IL-17-dependent invasiveness of breast cancer cell lines, we assayed the supernatants from IL-17-treated and control MDA-MB231 and MDA-MB453 cells for the presence of MMP-2, MMP-3, MMP-9 and TIMP-1. Control cells produced low levels of MMP-3 and MMP-9 (Figure 5a). Following treatment with TNFα, these levels were markedly increased. Despite the addition of up to 400 ng/ml recombinant IL-17, however, no increase in MMP-3 or MMP-9 was observed. The levels of MMP-2 released by breast cell lines (IL-17-treated or otherwise) was below the minimum sensitivity of the assay (data not shown). We measured the presence of the tissue inhibitors TIMP-1/TIMP-2 on the basis that decreased TIMP levels permit enhanced MMP function to be manifested. The level of soluble TIMPs, however, remained unchanged by the addition of IL-17 or TNFα (data not shown).

### IL-17 does not induce TNFα secretion from breast cancer cells

Several studies have highlighted an association between IL-17 expression and TNFα production, and in some systems IL-17 directly stimulates release of TNFα [28,30]. We therefore investigated the possibility that IL-17 exerted its effect on breast cancer cells by inducing TNFα secretion, which in turn could drive MMP-dependent tumour invasion. The breast tumour cell lines studied constitutively secreted low levels of TNFα, and this was not increased by the addition of 200 ng/ml IL-17 (Figure 5b). Furthermore, the addition of recombinant IL-17 did not increase the secretion of TNFα, irrespective of the dose of IL-17 added (data not shown). The levels of TNFα secreted following IL-17 treatment was in fact lower than control cells. One should bear in mind that these levels are very low, however, and variation at these levels is unlikely to be biologically significant. In this regard, using the TNFα ELISA we assayed the recombinant TNFα added as a positive control for the invasion assays (expected 40 ng/ml) and obtained a value of 17,205 ± 1,020 pg/ml. Therefore IL-17 does not appear to act by inducing TNFα production from breast tumour cells, and as such may represent an additional pathway involved in the spread of breast cancer.

## Discussion

The recent identification of a new subtype of T-helper cells (Th17) has prompted renewed interest in IL-17 biology. Our current understanding of IL-17 is that it plays an important role in inflammation, and is critical in host defence against infectious disease and in allergy and autoimmunity [33-35]. Most recently Hsu and colleagues reported that IL-17 from Th17 cells promotes the development of germinal centre-derived autoantibodies in a mouse model disease setting, due to the stimulatory effect on B-cell development [36]. Our data from control tonsil sections may also provide support for the concept that IL-17 plays an important role in B-cell development. In contrast to the murine model, however, our studies revealed that IL-17 expression was largely restricted to macrophages rather than T cells. It is worth emphasising that most studies to date are based on murine models, with a strong focus on Th17 cells. Although IL-17 is also expressed by epithelial cells, synovial cells and macrophages [1,37,38], the mechanisms driving IL-17 expression in these cell types remain unclear.

Pronounced leukocyte infiltration is considered a poor prognostic factor in breast cancer and those patients with tumoural leukocyte infiltration have a decreased 5-year survival [11,12]. Whilst the precise mechanisms for this remain unknown, there is a growing body of evidence to support the hypothesis that inflammation assists in the invasion of tumour cells [18-21]. This assistance involves the upregulation of IL-1, IL-6, IL-8, TNFα and MMPs that promote surrounding tissue destruction, leading to tumour invasion and ultimately metastasis. To our surprise, in our study there was little evidence of T cells expressing IL-17 in the tumours of breast cancer patients. In fact the majority of IL-17-positive cells (>98%) were macrophages, as defined by cytological criteria and CD68 staining. When we examined the stained tonsil tissue, which enlarges and becomes hyperplastic in response to infectious or allergic stimuli, we found abundant IL-17-positive cells located mainly in the germinal centres of the lymphoid follicles and in interfollicular areas. As in the breast tumours, the oval or irregular shape and the kidney-shaped nucleus that has a prominent nucleolus located at the nuclear membrane are the histopathological features that together with CD68 positivity enabled us to identify them as macrophages. Our finding together with the study of Fujino and colleagues – who reported IL-17-positive cells from inflammatory bowel disease, and found that most of the IL-17-positive cells are CD6-positive with a small number of CD3-positive cells [1] – indicates that IL-17-positive macrophages exist in some inflammatory conditions.

In addition to IL-17A, the subject of the current study, macrophages are also reported to express IL-17F, which is understood to suppress angiogenesis [39]. We propose to determine the relative expression of IL-17A versus IL-17F molecules in future studies. It has previously been shown that multinucleate giant cells express high levels of chemokines and MMP-9, and play an important role in inflammatory response [40,41]. The factors controlling multinucleate giant cell formation in cancer remain poorly understood, but a role for inflammatory cytokines such as IL-17 should not be discounted.

Whilst current theory supports the concept of inflammation promoting tumour progression, this is by no means the only possibility – and in breast cancer a body of evidence exists to oppose this. The significance of the inflammatory infiltrate in invasive carcinoma of the breast may represent an immune response to the tumour or may have protumour effects. Some large studies using multivariate analysis including grade suggested that inflammation is associated with better prognosis [42,43]. Nevertheless, we show that IL-17 promotes tumour invasion, and IL-17-positive macrophages were only identified patients with grade III disease. This may indicate a protumour effect of inflammation.

Co-culture of macrophages with breast cancer cells results in a marked increase in tumour cell invasion, which is associated with upregulation of MMP-2 and MMP-9 [44]. This invasion was attenuated by anti-TNFα antibodies and demonstrates that cancer cells utilise surrounding cells to facilitate invasion of the surrounding extracellular matrix. This represents a key mechanism by which tumour-infiltrating macrophages play an important role in cancer progression. The signals governing IL-17 expression by macrophages remain to be identified. Using tumour–macrophage co-culture systems, we are currently investigating the regulation of IL-17 expression by macrophage. Furthermore, we are examining the control of macrophage IL-17 expression by factors known to regulate the evolution of Th17 responses including IL-1, IL-6, IL-21 and transforming growth factor beta.

It should be noted that although MDA-MB435 is currently used in many studies as a breast cancer cell line, there is debate over its origin as one study suggests that this cell line is of melanoma origin [45]. In this regard we are currently expanding our work to include malignant melanoma, colorectal carcinoma, pancreatic carcinoma and ovarian carcinoma. We have begun staining a large series of whole sections from consecutively recruited breast cancer patients in Nottingham, UK. All of these patients have full follow-up data, so upon completion of the staining it will be possible to examine the correlation of IL-17 expression with patient parameters including lymphovascular invasion, stage, grade, 5-year survival, time to progression, and so forth [46].

IL-17-dependent invasion of breast tumours was blocked by a range of selective MMP antagonists for MMP-2, MMP-3 and MMP-9 as well as a broad-spectrum MMP antagonist. As we were unable to measure changes in MMP-2, MMP-3, MMP-9 or TIMPs following IL-17 exposure, however, we are unable to confirm that IL-17 acts through MMPs. In contrast, tumour cells secreted abundant MMP-3 and MMP-9 following TNF treatment, suggesting the use of nonredundant pathways by IL-17 and TNF. Whilst the exact mechanism of IL-17-dependent tumour cell invasion remains unknown, the antagonists we employed do not exclusively inhibit MMPs and may act on other classes of protease – including disintegrin and metalloproteinase proteins (for example, ADAM12) and astacins such as meprin, which displays activity similar to the gelatinases, MMP-2 and MMP-9 [47]. This will be investigated by gene expression studies with the use of more specific inhibitors and will be confirmed with siRNA. One possibility is that IL-17 acts through TNFα, but we saw no evidence of increased TNFα release from IL-17-treated tumour cells. There are several other candidates that may mediate the effect of IL-17. It is already known that IL-17 stimulates tumour cells, epithelial cells and fibroblast cells to produce IL-8 [48]. In turn, IL-8 promotes granulocyte recruitment and stimulates expression MMPs, remodelling the extracellular matrix and promoting cancer cell invasion [49]. In this regard we are presently investigating the regulation of factors such as IL-6, IL-8 and IL-22 in breast cancer cells treated with IL-17. Furthermore, we are presently investigating the expression of the two key IL-17 receptor chains on breast cancer cells, RA and RC, which heterodimerise to mediate signalling following binding of IL-17A and IL-17F.

## Conclusion

In summary, the present article is the first description of IL-17 protein expression in the context of human tumours and we have shown that expression appears largely restricted to macrophages. Most importantly, in companion experiments IL-17 directly induced breast cancer cell invasion independently of TNFα. Whilst invasiveness was inhibited by MMP selective antagonists, there were no measurable changes in levels of MMP-2, MMP-3 or MMP-9, raising the possibility of other classes of protease being involved. As such we describe a novel potential mechanism for breast cancer invasion and tumour progression. A detailed understanding of the IL-17-invasion axis may be of future prognostic and therapeutic value.

## Abbreviations

BSA: bovine serum albumin; ELISA: enzyme-linked immunosorbent assay; FCS: foetal calf serum; IL: interleukin; MEM: modified Eagle's medium; MMP: matrix metalloproteinase; PBS: phosphate-buffered saline; siRNA: small interfering RNA; Th17: T-helper cells type 17; TIMP: tissue inhibitor of matrix metalloproteinase; TNF: tumour necrosis factor.

## Competing interests

The present work was funded by grants from the University of Nottingham, Cancer Research Nottingham and the Institute of Infection, Inflammation and Immunity. These organisations will not gain or lose financially from the publication of this manuscript now and in the future. The University of Nottingham financed this manuscript. There are no nonfinancial competing interests to declare in relation to this manuscript.

## Authors' contributions

XWZ carried out the design of the study, the acquisition, analysis and interpretation of data, as well as drafting and revising the manuscript. LAM, AY and HAF carried out staining and *in vitro *experiment acquisition and analysis. RAAM helped with immunohistochemical interpretation, and provided critical comments on the manuscript. IOE, RAAM and AHSL undertook the histopathological scoring and data interpretation. LK carried out data acquisition and analysis. ECP carried out breast cancer section preparation and data coordination. IOE and HAF assisted with data interpretation and critically revised the manuscript. PMP and AMJ are the project coordinators and carried out the study design, data interpretation and manuscript revision.
